# An Intelligent Mental Health Identification Method for College Students: A Mixed-Method Study

**DOI:** 10.3390/ijerph192214976

**Published:** 2022-11-14

**Authors:** Chong Li, Mingzhao Yang, Yongting Zhang, Khin Wee Lai

**Affiliations:** 1 Graduate School, Xuzhou Medical University, Xuzhou 221004, China; 2 Institute of Medical Information Security, Xuzhou Medical University, Xuzhou 221004, China; 3 Department of Biomedical Engineering, Faculty of Engineering, Universiti of Malaya, Kuala Lumpur 50603, Malaysia

**Keywords:** facial emotion recognition, mixed method, deep learning, transfer learning, mental health

## Abstract

Purpose: Mental health assessments that combine patients’ facial expressions and behaviors have been proven effective, but screening large-scale student populations for mental health problems is time-consuming and labor-intensive. This study aims to provide an efficient and accurate intelligent method for further psychological diagnosis and treatment, which combines artificial intelligence technologies to assist in evaluating the mental health problems of college students. Materials and Methods: We propose a mixed-method study of mental health assessment that combines psychological questionnaires with facial emotion analysis to comprehensively evaluate the mental health of students on a large scale. The Depression Anxiety and Stress Scale-21(DASS-21) is used for the psychological questionnaire. The facial emotion recognition model is implemented by transfer learning based on neural networks, and the model is pre-trained using FER2013 and CFEE datasets. Among them, the FER2013 dataset consists of 48 × 48-pixel face gray images, a total of 35,887 face images. The CFEE dataset contains 950,000 facial images with annotated action units (au). Using a random sampling strategy, we sent online questionnaires to 400 college students and received 374 responses, and the response rate was 93.5%. After pre-processing, 350 results were available, including 187 male and 153 female students. First, the facial emotion data of students were collected in an online questionnaire test. Then, a pre-trained model was used for emotion recognition. Finally, the online psychological questionnaire scores and the facial emotion recognition model scores were collated to give a comprehensive psychological evaluation score. Results: The experimental results of the facial emotion recognition model proposed to show that its classification results are broadly consistent with the mental health survey results. This model can be used to improve efficiency. In particular, the accuracy of the facial emotion recognition model proposed in this paper is higher than that of the general mental health model, which only uses the traditional single questionnaire. Furthermore, the absolute errors of this study in the three symptoms of depression, anxiety, and stress are lower than other mental health survey results and are only 0.8%, 8.1%, 3.5%, and 1.8%, respectively. Conclusion: The mixed method combining intelligent methods and scales for mental health assessment has high recognition accuracy. Therefore, it can support efficient large-scale screening of students’ psychological problems.

## 1. Introduction

The special survey “Postgraduate Mental Health Status and Influencing Factors in 2019” [1] reported that college students are bothered with mental health problems due to academics, employment, research, interpersonal relationships, and other factors. A total of 35.5% of the students have a certain degree of depression, and 60.1% have anxiety and mental health problems. Similarly, *Nature Biotechnology* reported that the population of students with depression and anxiety is as much as six times that of the general population [2], and, especially during the time of COVID-19 [3,4,5], its population is still expanding. However, because college students have little knowledge of mental health or are concerned about gossip from their surroundings, it is difficult to detect mental health problems in this group on time.

To identify potential mental health problems accurately among college students, psychologists have proposed various methods, including observational, product analysis, experimental, and survey methods [6]. Among them, the observation method is to obtain the data required by systematically observing and examining the research object’s appearance, behavior, and language characteristics. The literature [7] has found that the proportion of college students suffering from mental health problems is usually higher than those with jobs. The product analysis method is an analysis method that analyzes the patient’s work through clinical symptoms and other materials to evaluate their psychological level and psychological status effectively. It is often used in the assessment of the psychological level of minors [8]. The experimental method is to evaluate mental health through comparative experiments. For example, Takarada explored the influence of the rotational training system on the mental health of Japanese dental graduate students by setting up a control group and an experimental group [9]. The investigation method is one of the most commonly-used methods, and it obtains the psychological activities of the subject by writing [10,11,12,13] or verbal answering questions. Byrom explored the relationship between population, individual background factors, stress, and mental health among 431 doctoral students [14]. Forbes participated in the study of 15 students employed in Queens Lanzhou in Queensland and analyzed the interviews to explore the factors relevant to the mental health of primary doctors in Australia [15]. Psychologists used standardized psychological test scales or complex instruments to assess subjects’ mental health problems [16,17]. Nisar used the Baker anxiety and depression Table to investigate the postgraduates of the Armed Forces in Lavar [18]. However, observation, work analysis, and experimental methods require much time to implement cumbersome processes. In addition, the work analysis method requires the researcher to provide the work. In contrast, the investigation methods are widely used in large-scale mental health diagnoses because of their simplicity, reasonable time scale, and excellent results.

Currently, the scale test is the primary method of screening psychological problems in universities. However, due to the deliberate concealment of some college students, its objectivity and accuracy are difficult to guarantee. Therefore, researchers propose to use artificial intelligence technology to solve the problem of mental health assessment and predict mental health based on physiological data, such as the electroencephalogram (EEG), facial emotion, voice, and skin electricity, using machine learning methods [19,20]. For example, Fei presented a novel deep convolution network-based emotion analysis framework to support mental state detection and diagnosis [21]. Wang classified the change in the eyes, eyebrows, and mouth angles, and constructed the early predictive model of depression [22]. Zhao described a deep learning approach that combined unsupervised learning, knowledge transfer, and hierarchical attention for the task of speech-based depression severity measurement [23]. However, the above methods all focus on model construction, which is directly related to the input characteristic data. They lack scales and other methods related to mental health assessment for comprehensive analysis. There are uncertainty problems for large-scale screening of students’ psychological problems.

It is challenging to consider high efficiency and accuracy when screening students’ psychological problems on a large scale. Therefore, we proposed a mixed-method study for intelligent mental health recognition. The method combines a psychological scale and a facial emotion recognition model. Among them, the Depression Anxiety and Stress Scale-21 (DASS-21), widely used to measure the mental health of Chinese university students, was selected as the scale for mental health assessment. We also designed a neural network-based facial emotion recognition model to capture the facial emotions of college students during the test. In addition, the model was pre-trained using a migration learning-based approach before practical application to optimize the emotion recognition results. Moreover, the model pre-training data were from both FER2013 and CFEE datasets. The FER2013 dataset consisted of 48 × 48-pixel face gray images, a total of 35,887 face images. The CFEE dataset contained 950,000 facial images with annotated action units (au). The experimental dataset was distributed online using a random sampling method. After matching the subjective evaluation reports, the proposed method obtained the complete mental health evaluation results for different facial features recognition. The test results showed that the proposed method had high accuracy in assessing the mental health of college students. It also had high efficiency because the facial emotion data were collected while answering the questionnaire.

## 2. Materials and Methods

### 2.1. Datasets

The FER2013 dataset [24] was contributed by Pierre-Luc Carrier and Aaron Courville, which consisted of 48 × 48-pixel grayscale images of faces, with a total of 35,887 facial images that have been registered. Our facial emotion recognition algorithm classified each emotion into seven categories: angry, disgust, fear, happy, sad, surprise, and neutral (The corresponding number was 0, 1, 2, 3, 4, 5, 6). The CFEE dataset was contributed by C. F. Benitez-Quiroz et al. [25] in 2017, totaling 950,000 pictures. These pictures contained basic emotions, composite emotions, and annotations of the emoticons.

We integrated the two datasets above for model training with more than one million samples. The training set ratio and test set were 7:3. Each training sample contained two columns, “Sentiment” and “Pixel”. The sentiment column contained a numeric code between 0 and 6 inclusive, representing the emotion in the image. The pixel column contained a quoted string for each image. The contents of this string were space-separated pixel values in row-major order. Each test sample contained only the “Pixels” column, and the task was to predict the “Sentiment” column.

### 2.2. The Sample Description

Participants were college students from Xuzhou Medical University who had been on campus since the COVID-19 outbreak. The recruitment group was approximately 350 students from the classes of 2020–2022, including 187 male and 163 female students with an average age of 22 years. Through a random sampling strategy, 400 online questionnaire emails were sent, and 374 responses were received; the response rate was 93.5%, and 350 questionnaires were available after data pre-processing. The recruitment period was April 2022. Exclusion criteria were whether they were students of a different academic year at the university.

### 2.3. The Recruiting Procedure

The students’ facial emotion data were collected during the online questionnaire test, and then the data were pre-processed to remove outliers, null values, and other outliers. The experiment data included 350 pieces of data from187 boys and 163 girls.

The pre-trained model was used for emotion recognition and given the comprehensive psychological assessment score by arranging the scores of the online psychological survey questionnaire and facial emotion recognition model. Finally, the single psychological questionnaire survey and the comprehensive psychological evaluation results were compared.

### 2.4. Mental Health Assessment

This study uses the simplified Chinese version of DASS-21 revised, and the validity and reliability indices of the DASS-21 scale are available through citations [26]. Initially, The Depression Anxiety and Stress Scale (DASS) was originally proposed by Lovibond [27]. It draws on the architecture of the tripartite model [28], defining the scale as a three-dimensional structure, where variables are just depression, anxiety, and stress. There are 21 items on the scale used in this study and seven on each of the three subscales of depression, anxiety, and stress. The 4-level scores are scored on a scale of “0” (disagree), “1” (somewhat), and “2” (frequently) to “3” (always). The score of each subscale is multiplied by 2 to achieve the subscale score. The higher the score, the more emotional. The test results of the three subscales are graded, as shown in Table 1. According to the literature [29], all items on the DASS-21 are summed to measure overall distress (ranging from 0 to 63).

### 2.5. Facial Emotion Recognition Model

Facial emotions that are not easily changed by human control can reflect people’s physical health and psychological emotions [30]. Psychologists perform psychological counseling and give treatment recommendations by observing people’s facial emotions and physical activity. Therefore, we combine psychological questionnaires with facial emotion analysis to comprehensively evaluate students’ mental health. A facial emotion recognition algorithm called FER-CNN is proposed to capture the emotions of college students, achieving high-quality data of the extracted features of facial emotions. In addition, transfer learning [31] is used to reduce the reliance on facial physics-based models and other pre-processing techniques. We use a pre-trained model classifier to determine facial emotions based on one of the facial categories, extract spatial and temporal features from facial components and landmarks, and convolve the input image with filters in convolutional layers based on the convolution results, and feature maps are constructed, and max-pooling (subsampling) layers reduce the spatial resolution of a given feature map. Convolutional Neural Network (CNN) applies a fully connected neural network layer after the convolutional layer and recognizes individual facial emotions based on the output of the activation function SoftMax.

#### Classifiers

CNN employed in this study has nine neural network layers, including four convolutional layers, three pooling layers, and two fully connected layers. We use a pre-trained model undertraining more than one million images. Using a pre-trained CNN as a deep feature extractor requires extracting deep features by the above pre-trained CNN model. On this basis, we use a transfer learning strategy to reduce the training time of the network. The output of the activation function forms the neurons of the current layer, which form the feature map of the current convolutional layer. The following function can describe the calculation:

(1) $$X_{j}^{l}=F(\sum_{i\in M_{j}}X_{i}^{l-1}*w_{ij}^{l}+w_{b})$$

where $X_{i}^{l-1}$ is the feature map output from the *L* − 1 layer, ∗ represents the convolution operation, and $w_{ij}^{l}$ and $w_{b}$ represent the weight and bias, respectively.

Each convolutional layer is followed by a Rectified Linear Unit (ReLU) layer to increase the nonlinearity of the network. There are two reasons for using ReLU. On the one hand, ReLU is a half-wave rectifier function, and it can reduce training time while preventing overfitting. On the other hand, ReLU prevents the vanishing gradient, and it can run faster than other logistic functions. The ReLU layer for input x can be described as:

(2) F(x)=max(0,x)

There is also a max-pooling layer after the convolutional layer, which is used for down-sampling. On the one hand, the down-sampling process does not change the number of feature maps. On the other hand, the down-sampling process removes unnecessary information and reduces the number of parameters of feature maps. The down-sampling layer can be described as:

(3) $$X_{j}^{l}=F(\mathrm{down}(X_{j}^{l-1})+w_{b})$$

where $X_{j}^{l}$ represents the *j* th feature map of pooling layer *l*, and $w_{b}$ represents the offset term of the down-sampling layer.

Finally, after the four convolutional layers, the model has two fully connected layers, which is considered a convolutional layer. In addition, its kernel size and input data size should be consistent with those used in convolutional layers. The fully connected layer can be described as:

(4) $$X_{j}^{l}=F(\sum_{i}w_{ij}^{l}X_{i}^{l-1}+w_{b}^{l})$$

When recognizing facial emotions online, there is no need to retrain the model, and it only extracts deep features, which can quickly and accurately realize online recognition.

### 2.6. Comprehensive Mental Health Score

**Figure 1:**
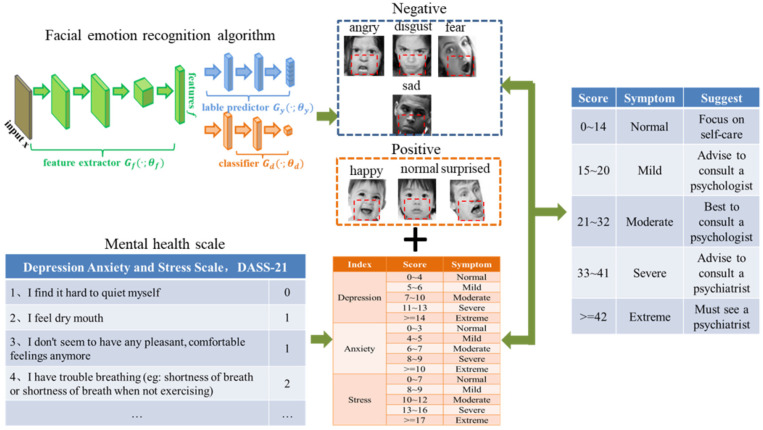
Full text framework.

After an online questionnaire survey and facial emotion recognition, as shown in Figure 1, we obtained written reports and emotion scores. Then, a comprehensive evaluation index provided more accurate psychological assessments and treatment suggestions, as shown in Table 2. The primary process was as follows:

Use the facial emotion recognition model to identify the participants’ emotional features during the evaluation process and divide the identified emotions into positive and negative categories.

The various emotional traits displayed by the participants were summarized in time series, and the three mental health traits involved in the questionnaire (including stress, anxiety, and depression) were assessed by establishing a questionnaire. According to the results of the facial emotions, the participants’ mental health assessment level was revised to obtain their mental health status as much as possible.

## 3. Results

### 3.1. The Proposed Model Performance Evaluation

**Figure 2:**
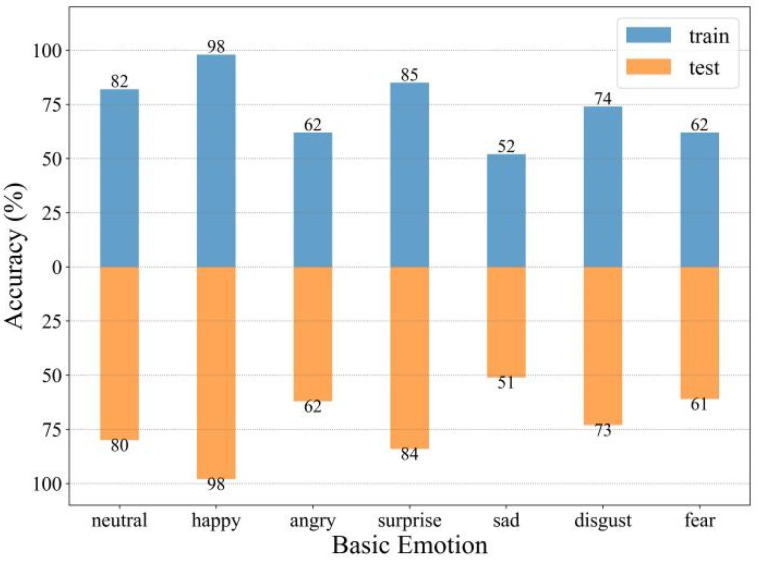
Accuracy of seven kinds of facial emotional recognition.

To evaluate the performance of the proposed facial emotion recognition algorithm, we used FER2013 and CFEE datasets to train the facial emotion recognition model with 790,120 pictures and tested the facial emotion recognition model with 295,766 pictures, obtaining the accuracy of detecting seven facial emotions. The results can be seen in Figure 2.

The training accuracies of normal, happy, angry, surprise, sad, disgust, and fear emotions were 82%, 98%, 62%, 85%, 52%, 74%, and 62%. The test accuracies of normal, happy, angry, surprise, sad, disgust and fear emotions were 80%, 98%, 62%, 84%, 51%, 73%, 61%. The proposed facial emotion recognition algorithm had a high detection effect on seven facial emotions.

**Figure 3:**
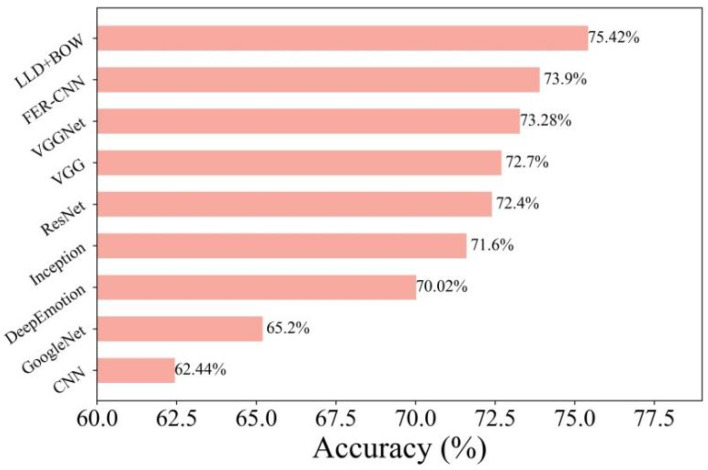
Comparison of the SOTA technique and FER-CNN algorithm based on the FER2013 dataset.

In addition, we experimented with the State Of The Art (SOTA) technique based on the FER2013 dataset to verify the practicality of the FER-CNN algorithm. The SOTA technology used in the experiment included CNN [32], GoogleNet [33], DeepEmotion [34], Ineeption [35], ResNet [35], VGG [35], VGGNet [36] and LLD+BOW [37]. The results can be seen in Figure 3.

The accuracy of CNN, GoogleNet, DeepEmotion, Inception, ResNet, VGG, VGGNet, FER-CNN and LLD+BOW was 62.44%, 65.2%, 70.02%, 71.6%, 72.4%, 72.7%, 73.28%, 73.9% and 75.42%. The algorithm with the best performance was LLD+BOW, followed by FER-CNN. The accuracy of FER-CNN was 1.52% lower than LLD+BOW. Compared with several other algorithms, the maximum accuracy difference was 11.46%. The FER-CNN algorithm still had a large advantage. Therefore, the proposed FER-CNN algorithm has strong practical application value in facial emotion recognition.

Furthermore, we compared the proposed FER-CNN algorithm with the Derl algorithm [38] on the Fer2013 and CFEE datasets to verify the robustness of the FER-CNN algorithm. The results can be seen in Table 3.

On the Fer2013 dataset, the training accuracy difference between FER-CNN and Derl algorithm was small, only 5.3%. The test accuracy difference reached 21.9%, and the FER-CNN algorithm had a stronger detection ability. On the CFEE dataset, the training accuracy difference between FER-CNN and Derl algorithm was 7.5%, and the test accuracy difference was 4.8%. The detection ability of the FER-CNN algorithm was still higher than that of the Derl algorithm.

In summary, the FER-CNN algorithm outperforms the Derl algorithm. It has higher facial emotion recognition accuracy, mainly due to the use of transfer learning to train the model, so that before fine-tuning, the model’s initial performance is higher, and the rate of model improvement is faster during the training process. In addition, the performance of the FER-CNN algorithm is also robust and not limited to a single dataset.

### 3.2. Comprehensive Mental Health Assessment

**Figure 4:**
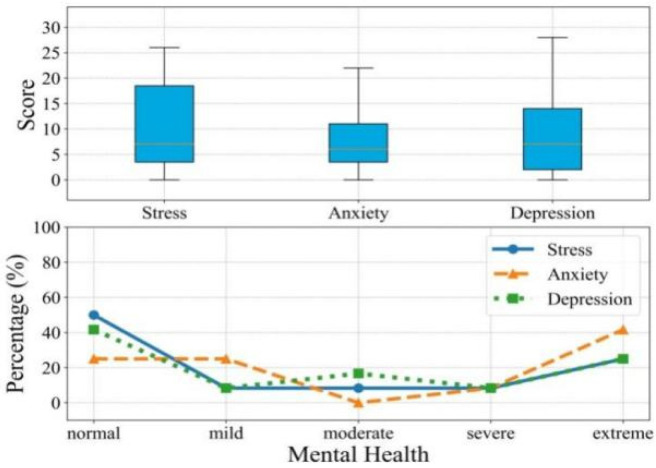
Distribution of mental health problems. (**Top**) Boxplots of the distribution of scores for the three mental states of the participants; (**Bottom**) The proportion of the number of participants according to the severity of the three psychological problems measured by the participants.

The FER-CNN algorithm captured students’ facial emotions in real-time and gave evaluation results. A comprehensive psychological assessment score was given by arranging the scores of the online psychological survey questionnaire and facial emotion recognition model. The single psychological questionnaire survey and the comprehensive psychological evaluation results were compared. The results can be seen in Figure 4.

As shown in Figure 4, in the three subscales of depression, anxiety and stress, 41.67%, 25% and 50% of students showed normal symptoms, 8.33%, 25%, 8.33% showed mild symptoms, 16.67%, 0%, 8.33% showed moderate symptoms, 8.33%, 8.33%, 8.33% showed severe symptoms, and 25%, 41.67%, 25% showed extreme symptoms. Overall, most students have different degrees of mental health problems. However, this result was inaccurate because some students may have concealed or reported personal information incorrectly.

**Figure 5:**
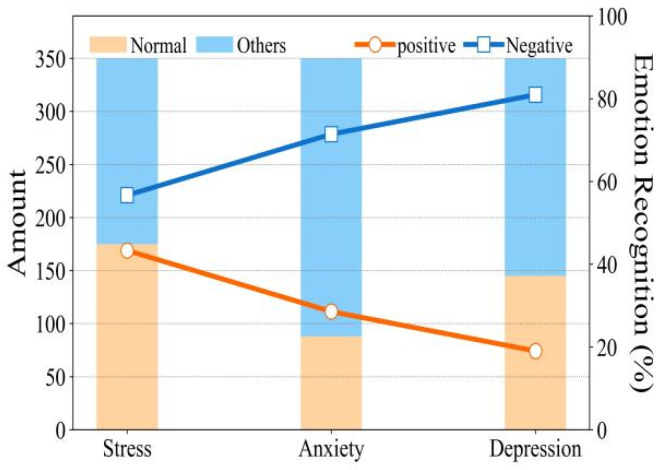
Correspondence map between facial emotion recognition and mental health score.

To improve the detection accuracy of the mental health survey questionnaire, we applied the facial emotion recognition model to the questionnaire survey process. The experimental results are shown in Figure 5.

The bar graph shows the scores of the mental health survey questionnaire of 350 students, and the line graph shows the proportion of negative and positive emotions recognized by the facial emotion recognition model during the questionnaire survey. The proportions of depression, anxiety, and stress symptoms were 58.33%, 75%, and 50%, respectively. The recognition ratios of negative emotions in the three symptoms were 81%, 71.4%, and 56.7%. The results show that the more serious the psychological problem is, the higher the ratio of negative emotion recognition is. Mental health recognition is related to positive and negative emotion recognition.

Table 4 presents the results of the single mental health questionnaire and the comprehensive facial emotion recognition model for depression, anxiety, stress, and combined score. In addition, the results were compared with the results of a large-scale mental health survey of students of the Chinese Academy of Sciences [1].

Compared with the results of the large-scale mental health survey, the three symptoms of depression, anxiety, and stress in the mental health questionnaire and the absolute value of the scoring error of the combined score were 10.8%, 14.9%, 23.5%, and 8.9%, respectively. Additionally, the absolute values of the scoring error of the comprehensive facial emotion recognition model were 0.8%, 8.1%, 3.5%, and 1.8%, respectively. The comprehensive facial emotion recognition model performed better than the single mental health questionnaire. The results are very close to those of the large-scale mental health survey. Our comprehensive model also divides the test results into more detail and gives further recommendations, which can accurately and efficiently detect students’ mental health problems and avoid a series of dire consequences.

## 4. Discussion

With the increase in college students’ psychological problems year-on-year, Colleges and universities at home and abroad have paid attention to large-scale normalized psychological problems. However, traditional psychological diagnostic methods, such as observation, work analysis, and experimental, are time-consuming and labor-intensive. Moreover, the concealment of students and the incomplete coverage of questionnaire questions may cause serious misdiagnosis and missed diagnosis. As mental health is related to emotions, explaining mental health problems by observing facial emotions is a hot topic of extensive research by scholars [39,40,41], for example, C. Priyesh [42] performed facial emotion recognition in patients with schizophrenia and bipolar disorder with low recognition accuracy and reaction time. The experimental results illustrate the correlation between mental illness and facial emotions. Fava, M. et al. [43] suggested that irritability in the student population may indicate a major depressive disorder. The above study explored the relationship with mental health by artificially observing the emotions of the study participants, which was laborious and a significant waste of resources. In our study, we use machine learning to identify the facial emotions and psychological scales to measure the mental health of the study subjects, which saves time and effort and has high identification efficiency. Bilgi, M.M. et al. [44] explored the relationship between schizophrenia and emotion recognition using the Irritability Questionnaire and the Facial Emotion Recognition Method. All these studies have demonstrated the effectiveness of our method [45]. However, the proposed method also has some limitations, as it identifies students’ facial emotions, which will not be recognized if the camera is turned off or the students’ faces are obscured. The problem can be solved by calculating the face share and issuing a pop-up to alert the research subject if the face share is lower than the set threshold.

The method proposed in this paper is an excellent auxiliary tool for college students’ mental health identification, which can identify students’ mental health problems more comprehensively and provide extra data for later clinical diagnosis. In addition, the use of transfer learning to pre-train the model can achieve a high accuracy rate in the identification process, which is especially suitable for large-scale routine mental health screening of college students. Another potential use of this integrated model could be to explore the risk and timing of mental health episodes in students. Identifying changes in students’ mental health over time by tracking changes in patients’ moods is one of our future research directions.

## 5. Conclusions

This study proposes a mixed method that combines psychometric scales and facial emotion recognition models to achieve efficient and accurate mental health identification of college students. We precisely capture subjects’ emotional facial status in testing, characteristic types are acquired through facial emotion recognition technology, and emotions are classified into two categories, positive and negative. Among them, the higher the proportion of negative emotions, the more serious the mental health situation [30]. We use transfer learning to optimize the results of extracting data. The method proposed in this paper achieves a better mental health status assessment by combining the proportion of positive and negative emotions in the evaluation process after matching the subjective assessment report, giving a complete mental health evaluation result.

## Figures and Tables

**Table 1:** Three scale score classification table.

Index1	Score	Symptom
Depression	0~4	Normal
	5~6	Mild
	7~10	Moderate
	11~13	Severe
	≥14	Extreme
Anxiety	0~3	Normal
	4~5	Mild
	6~7	Moderate
	8~9	Severe
	≥10	Extreme
Stress	0~7	Normal
	8~9	Mild
	10~12	Moderate
	13~16	Severe
	≥17	Extreme

**Table 2:** Score grading and recommendations for comprehensive facial emotion recognition.

Score	Symptom	Suggest
0~14	Normal	Focus on self-care
15~20	Mild	Advise to consult a psychologist
21~32	Moderate	Best to consult a psychologist
33~41	Severe	Advise to consult a psychiatrist
≥42	Extreme	Must see a psychiatrist

**Table 3:** Comparison of facial recognition algorithms.

Methods	Fer2013		CFEE	
	*Train*	*Test*	*Train*	*Test*
FER-CNN	0.739	0.6298	0.814	0.779
Derl	0.686	0.4108	0.739	0.731

**Table 4:** Comparison of a single mental health questionnaire, a comprehensive facial emotion recognition model, and statistical results from a large-scale graduate student mental health survey.

Index	Survey Questionnaire	Comprehensive Evaluation Model	Large-Scale Survey Statistics
Depression	50%	60%	60.8%
Anxiety	75%	52%	60.1%
Stress	59%	39%	35.5%
Combine Score	61%	50.3%	52.1%

## Data Availability

The data analyzed in this study are available from the Institute of Medical Information Security, Xuzhou Medical University, but restrictions apply to the availability of these data, which were used under license for the current study, and so are not publicly available. Requests to access these datasets should be directed to Xiang Wu: lichong1985@xzhmu.edu.cn.
